# Reaction rate law model and reaction mechanism covering effect of plasma role on the transesterification of triglyceride and methanol to biodiesel over a continuous flow hybrid catalytic-plasma reactor

**DOI:** 10.1016/j.heliyon.2020.e05164

**Published:** 2020-10-09

**Authors:** P. Purwanto, Luqman Buchori, I. Istadi

**Affiliations:** Department of Chemical Engineering, Faculty of Engineering, Universitas Diponegoro, Jl. Prof. Soedarto, SH, Semarang 50275, Indonesia

**Keywords:** Chemical engineering, Energy, Organic chemistry, Catalyst, Chemical reaction engineering, Industrial chemistry, Biofuel, Fuel technology, Hybrid catalytic-plasma reactor, Reaction rate law model, Biodiesel, Fatty acid alkyl ester, Plasma roles

## Abstract

This study investigated predictions of reaction mechanisms and reaction rate law model covering effect of plasma role on the heterogeneous catalytic reaction of triglyceride and methanol to produce biodiesel (fatty acid methyl ester - FAME or fatty acid alkyl ester – FAAE) over a continuous flow hybrid catalytic-plasma reactor. This catalytic reaction was carried out in a dielectric-barrier discharge plasma reactor over 5 wt% K_2_O/CaO–ZnO catalyst under conditions of atmospheric pressure and the reactor temperature of 65 °C. During the hybrid catalytic-plasma reaction system, the voltage, the catalyst diameter, and the Weight Hourly Space Velocity (WHSV) were kept constant at 5 kV, 5 mm, and 1.186/min, respectively. It was found that transesterification reaction with the hybrid roles of catalytic and plasma achieved 77.2% biodiesel yield. Kinetic studies of this transesterification reaction over a continuous flow hybrid catalytic-plasma reactor suggested following Eley-Rideal mechanism model, where the methanol adsorbed on the catalyst surface reacted with triglycerides in bulk phase to produce an adsorbed methyl ester and glycerol in bulk phase. The possible reaction rate law model found is: -r_TG_ = r_ME_ = r_s_ = (0.0078∗(0.0061∗C_TG_∗C_M_^3^–3.0302 × 10^−6^∗C_ME_^3^∗C_G_))/(0.1827∗C_M_+ 0.0145∗C_ME_+1)^3^ gmol/gcat.min. This reaction rate law model was useful to design reactor of the hybrid catalytic-plasma chemical reaction system for biodiesel production.

## 1 Introduction

Biodiesel is an alternative fuel made by transesterification reaction of oil and alcohol. Biodiesel can be produced by conventional transesterification method, i.e. homogeneous, heterogeneous, and enzyme or advanced technology processes (microwave, ultrasound, or plasma-assisted process). Plasma technology in the field of chemical reactions is developing quite rapidly in addition to existing conventional technologies. There is a significant difference between heating with plasma technology and heating with conventional technology. In conventional heating, the required reaction temperature is quite high, whereas in plasma heating, the required temperature is quite low but can produce high energy electrons with a temperature of about 10^4^ K (Istadi et al., 2014), so that it is able to excite the components in the reactants. This is much different from conventional heating which has low energy in breaking bonds in the reactants (Fridman, 2008). Heating with plasma can significantly reduce the activation energy so that the reaction time can take place faster. Plasma-assisted technology has several advantages compared with a conventional method, such as: shorter reaction time, no soap product, no glycerol product, and higher biodiesel yield (Buchori et al., 2016a, Buchori et al., 2016b; Istadi et al., 2014).

**Figure 1:**
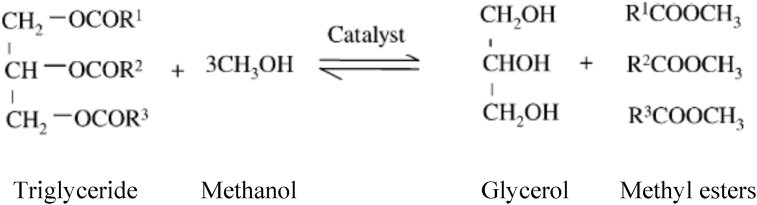
Transesterification reaction of triglyceride with methanol.

In the transesterification reaction, in the first step, triglyceride reacts with methanol to produce diglyceride. Furthermore, diglyceride reacts with methanol to produce monoglyceride. Finally, monoglyceride reacts with methanol to produce methyl ester and glycerol (Aransiola et al., 2013; Feyzi and Khajavi, 2016; Muazu et al., 2015; Muciño et al., 2016). By assuming that the reaction is a single step transesterification, the intermediate reactions of diglyceride and monoglyceride could be ignored (Birla et al., 2012; Kusdiana and Saka, 2001; Vujicic et al., 2010). Thus, stoichiometrically, the transesterification reaction requires 3 mol of methanol and 1 mol of triglycerides as illustrated in Figure 1.

Previously, plasma assisted technology has been developed by several researchers, including a plasma reactor for biodiesel production (Abdul-Majeed et al., 2016; Buchori et al., 2016a, Buchori et al., 2016b; Cubas et al., 2015; Hyun et al., 2012; Istadi et al., 2014; Lawson and Baosman, 2010). Plasma process without catalyst was developed by Lawson and Baosman (2010), Istadi et al. (2014), Cubas et al. (2015), and Abdul-Majeed et al. (2016). They found some difficulties on the controlling the reaction direction due to uncontrollable high energetic electrons from the plasma. Meanwhile, Hyun et al. (2012) and Buchori et al., 2016a, Buchori et al., 2016b developed a hybrid catalytic-plasma or plasma-assisted catalytic technology with the presence of catalyst to adjust the reaction direction. They found that the plasma-assisted transesterification process can produce high biodiesel yields, very fast reaction times, and easier product separation than conventional catalytic processes. However, further studies on the synergistic effects of plasma energetic electrons and catalyst roles on transesterification reactions need to be developed, especially on the kinetic studies due to plasma roles to support the reactor design that have not been developed by previous researchers. This is intended to investigate the extent to which the effectiveness of plasma and/or catalyst in the transesterification process and compared to the conventional catalytic process (without plasma).

The process of plasma-assisted technology in the transesterification reaction is influenced by several operating parameters, including WHSV, reaction time, and voltage. Buchori et al. (2017) found that FAME yields declined with increasing WHSV. The higher the WHSV, the shorter the contact time between high-energy electrons and reactant molecules, so the collision process will also be shorter. As a result, only a few electron covalent bonding pairs in the reactant molecule are excited. The reaction time required for plasma-assisted technology is very short. Research conducted by Buchori et al. (2017) only takes 1.25 min to produce a 77.19% FAME yield. The reaction time required for plasma-assisted technology is very short. Meanwhile, the voltage also affects the yield of FAME. The obtained FAME yield decreases because of the higher the voltage, the catalytic-plasma process produces unwanted chemicals.

Previous researchers suggested the transesterification reactions models i.e. homogeneous catalytic transesterification reaction models (Bambase et al., 2007; Freedman et al., 1986; Krishnan and Dass, 2012; Vicente et al., 2005) and heterogeneous catalytic transesterification models (Avhad et al., 2016; Kumar and Ali, 2013; Nambo et al., 2015). In the heterogeneous catalytic reactions, Feyzi and Khajavi (2016) and Portha et al. (2012) studied biodiesel synthesis using a heterogeneous catalyst and they found a second-order kinetic as the best kinetic modeling of experimental data. The found kinetic reaction rate models only appropriate for conventional heterogeneous catalytic reactions by considering mechanism of the reactions, i.e. conventional batch and fixed bed reactors (Al-Sakkari et al., 2017; Hsieh et al., 2010; Kapil et al., 2011; Xiao et al., 2010). The K_2_O/CaO–ZnO catalytic test on the hybrid catalytic-plasma reactor has indeed been carried out (Buchori et al., 2017). They tested the effect of catalyst basicity on the yield of FAME. In addition, they also compared the FAME yield when transesterification was carried out without plasma, with plasma and without a catalyst. However, we have not studied the kinetic models for the transesterification over the catalytic plasma reactor using the K_2_O/CaO–ZnO catalyst. The proposed novel reaction rate kinetic model includes the roles of plasma assistance on the transesterification process. The developed reaction rate model is important for designing and scaling up the continuous flow hybrid catalytic-plasma reactor on its implementation. Therefore, this paper focuses to study the development of a novel reaction rate law model appropriate to the continuous flow plasma-assisted catalytic transesterification reaction using a new solid base catalyst (K_2_O/CaO–ZnO). This proposed reaction rate law considers the effect of plasma role on assisting the catalytic transesterification process, as well as its predicted reaction mechanism.

## 2 Materials and method

### 2.1 Materials

Soybean oil, as raw material, was obtained from the local market. Methanol with analytical grade (99.9%, Merck) was used another reactant. The chemicals used for catalyst preparation are calcium nitrate tetrahydrate (Ca(NO_3_)_2_.4H_2_O) (99%, Merck), zinc nitrate hexahydrate (Zn(NO_3_)_2_.6H_2_O) (98.5%, Merck), potassium nitrate (KNO_3_) (99%, Merck), sodium carbonate anhydrous (Na_2_CO_3_) (99%, Merck), and sodium hydroxide (NaOH) (99%, Merck).

### 2.2 Catalyst preparation

The catalyst used, 5 wt% K_2_O/CaO–ZnO (5KCZ), was prepared by co-precipitation and impregnation methods. The molar ratio of Ca to Zn in the CaO–ZnO catalyst was 3:1. The CaO–ZnO catalyst was prepared by mixing solution of 2 M Ca(NO_3_)_2_.4H_2_O and 2 M Zn(NO_3_)_2_.6H_2_O, and was slowly dropped wisely 10 mL/min of 2 M Na_2_CO_3_ solution to form a gel-like substance. While vigorously stirred, a 1 M NaOH solution was added into the mixed solution until achieves pH 10. After stirred for 24 h at a temperature of 60 °C, the solid product was then filtered and washed by aquadest until alkali-free condition. The solid product was then dried in an oven (Memmert) at 110 °C for overnight and calcined in a box furnace (Ney Vulcan 3–550) at a temperature of 800 °C for 3 h and called as CaO–ZnO (CZ) catalyst. The 5KCZ catalyst was obtained by impregnating 0.5 M KNO_3_ solution on the resulted CaO–ZnO catalyst. The resulted materials was dried in the oven at 110 °C for overnight and thus calcined in the box furnace for 5 h at a temperature of 300 °C to produce 5 wt% K_2_O/CaO–ZnO catalyst. The resulted catalyst was crushed and then was pelleted into cylindrical pellets with averaged diameter (*d*_*p*_) of 5 mm. The catalyst pellet was made using a pelletizer tool. The crushed catalyst is then pressed into the hole in the center of the circular mold. This cylindrical hole, with a diameter of 5 mm and a thickness of 5 mm, was designated to form pellets. Next, the hole of the mold was filled with catalyst powder. The catalyst pellet was formed by pressing the catalyst-filled mold with a stainless-steel rod (5 mm diameter) from the pelletizer for 1 min. The catalyst was then removed from the hole to get a 5 mm diameter catalyst pellet.

### 2.3 Catalyst characterization

The catalyst was characterized by X-Ray Diffraction (Shimadzu XRD-7000) and its basicity is tested by titration method. XRD analysis was performed with Cu-Kα radiation at k = 1.54 Å operated at 30 mA and 30 kV. The scanning speed of the sample was 4°/min with 2θ angle ranges of 10^o^ to 90^o^. The diffractogram/peak was compared to the data from JCPDS (Joint Committee on Powder Diffraction Standards) library to determine the compounds of crystal in the catalyst.

The titration method for determining the basicity of the catalyst follows the method introduced by Tanabe and Yamaguchi (1964). Initially, the catalyst was crushed and sieved at 100–200 mesh. Next, a 0.5 g catalyst sample was mixed with 20 mL benzene. A total of 1 mL indicator solution consisting of 128 mg bromothymol blue (BTB) in 100 mL benzene is poured into the mixture. The color of the catalyst sample suspension changes from yellow to green blue. After that, 0.1 N benzoic acid is added dropwise into the catalyst suspension until the green color of the solid particles disappears. The end point of the titration is determined when all the green colors disappear. The basicity was calculated from Eq. (1).

(1) $$Basicity\left(\frac{mgrek}{g}\right)=\frac{{\left(VxN\right)}_{benzoicacid}}{W_{catalyst}}x100\%$$

where V is the volume of the benzoic acid solution (mL), N is the normality of the benzoic acid solution (mgrek/mL), and W represents the weight of the catalyst sample (g).

### 2.4 Catalyst testing for transesterification reaction process in a continuous flow hybrid catalytic-plasma reactor

**Figure 2:**
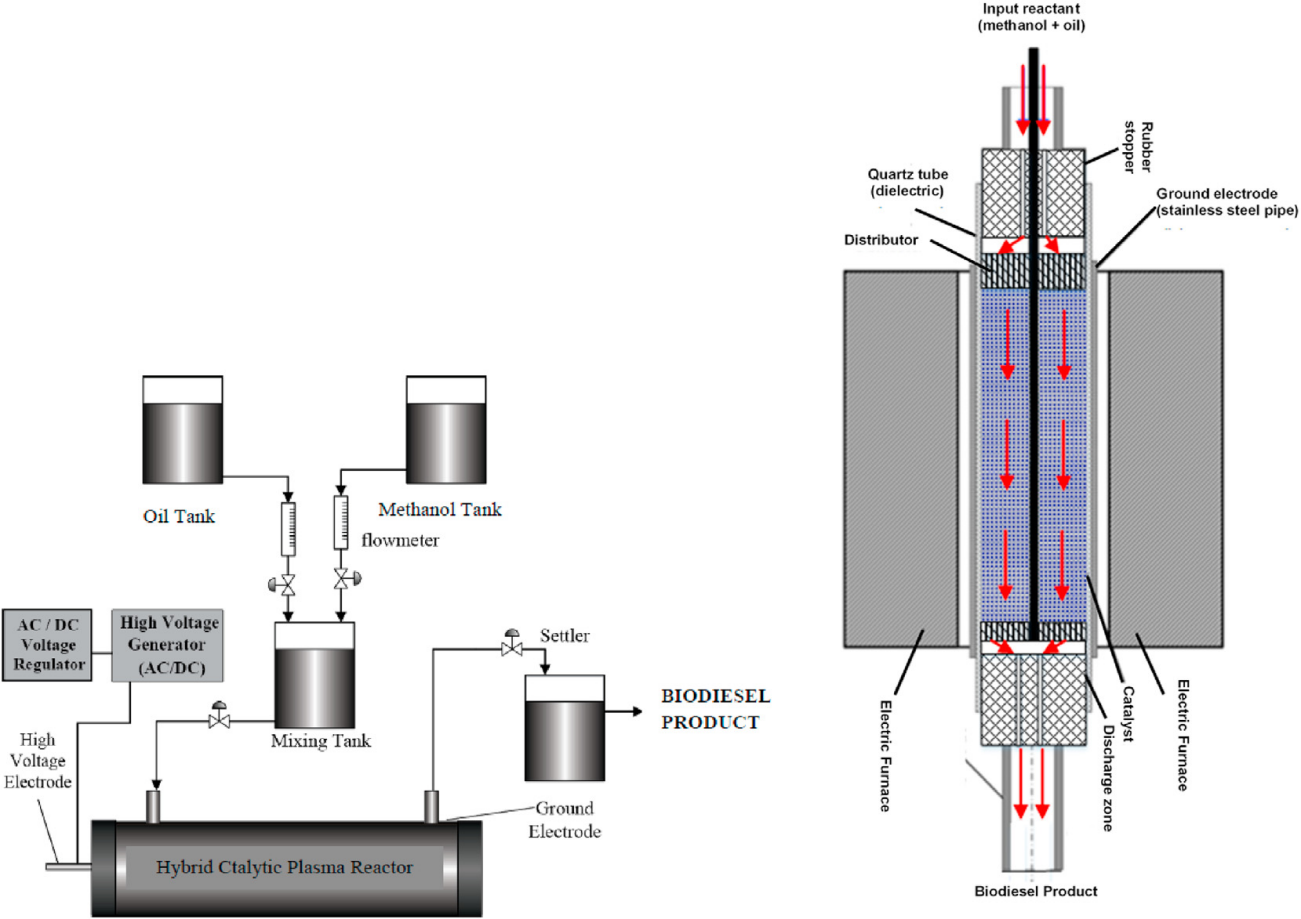
Transesterification process using hybrid catalytic plasma reactor.

Methanol and soybean oil, with molar ratio of methanol to oil as explained in Table 1, were inserted into the mixing tank and vigorously stirred. The mixed reactants were flowed into the reactor using a peristaltic pump. We varied mole of the triglycerides, whilst the mole of methanol was kept constant and vice versa, in order to study the dependence of vegetable oil (triglycerides) on reaction rate (-*r*_*TG*_), Transesterification reaction was carried out in a fixed catalytic-DBD (Dielectric Barrier Discharge) plasma reactor with 2.54 cm (1 inch) in diameter and 30 cm in length. The reactor was equipped by a ground electrode (stainless steel) on outside of glass reactor coaxially and a dielectric barrier discharge of glass material inside of the ground electrode coaxially, while a high voltage electrode made from copper rod was put inside the reactor axis. A coaxial space/gap between the high voltage rod and the glass dielectric barrier was called as a discharge zone. An amount of 15.15 g 5KCZ catalyst was inserted into the discharge zone within the reactor. This catalyst filled the reactor inside as high as 5.6 cm. The DBD catalytic-plasma reactor was heated up to 65 °C in an electric split tube furnace connected with a temperature controller to keep the temperature constant. The reactant mixture was then fed into the plasma reactor through a catalyst located within the discharge zone. A high voltage (0–12 kV) from the DC-type high voltage power supply was supplied to the the plasma reactor through the high voltage electrode copper rod. The high voltage electrode provided high energetic electrons to the ground electrode through the glass-based dielectric barrier discharge. The high energetic electrons collided with reactants molecules and catalyst material, leading to molecular effect on the catalyst and reactants molecules. The electrons flow and distribution were controlled and distributed by the glass dielectric barrier through the discharge zone. The discharge voltage was measured by a digital oscilloscope (Tektronix TBS 1052B-EDU 50 MHz 1 GS/s) through a high voltage probe (x1000). The biodiesel product was collected in a product tank and then analyzed by the Gas Chromatography-Mass Spectrometry (GC-MS) to determine the composition of fatty acid methyl/alkyl esters (FAME/FAAE). Figure 2 shows the process of transesterification reaction in a continuous flow hybrid catalytic-plasma reactor.

**Table 1:** The concentrations of reactant and product, and reaction rate of product in the transesterification reaction of biodiesel production in the hybrid catalytic-plasma reactor.

Run	Molar ratio		C_TG__Triglyceride_ (gmol/L)	C_M__Methanol_ (gmol/L)	C_ME__FAAE_ (gmol/L)	C_G__Glycerol_ (gmol/L)	$r_{FAAE}^{{\;}^{\prime }}\left(\frac{gmol_{FAAE}}{gcat.min}\right)$
	Methanol	Triglyceride					
1	3	1	0.910	2.729	0.271	0.588	3.617 × 10^−4^
2	5	1	0.847	4.237	0.319	0.468	4.268 × 10^−4^
3	7	1	0.793	5.551	0.505	0.433	6.744 × 10^−4^
4	17	1	0.601	10.208	1.034	0.447	13.795 × 10^−4^
5	1	0.5	0.944	1.888	0.517	0.719	6.899 × 10^−4^
6	1	1	0.982	0.981	0.657	0.503	8.771 × 10^−4^
7	1	2	1.002	0.500	1.171	0.650	15.624 × 10^−4^
8	1	3	1.008	0.337	0.473	0.761	6.406 × 10^−4^

### 2.5 Biodiesel product analysis

The reaction product was analyzed by a Gas Chromatography-Mass Spectrometry (GC-MS) (Shimadzu QP2010S) equipped with a DB-1 column. The GC-MS analysis was programmed by heating the sample at 50 °C oven temperature (held for 5 min) and ramped 10 °C/min to 260 °C and held for 33 min at this temperature.

### 2.6 Analysis of Thiele Modulus and effectiveness factor to maintain the surface reaction rate limiting step mechanism

The reaction rate law model of a reaction is valid when the catalyst surface reaction mechanism control the overall reaction rate. This condition is achieved when the catalyst particle size is not exceeding the certain maximum limit formulated by Weisz-Prater criterion (Fogler, 2006) or when kept within surface reaction limited (not internal/external diffusion limited). Weisz-Prater criterion were analyzed by calculating the relation of effectiveness factor and Thiele modulus. For pellet-shaped catalysts, the effectiveness factor is calculated using Eq. (2) with assumption that the transesterification reaction followed the first order reaction due to excess condition of one of the reactants:

(2) $$\eta =\frac{3}{\phi_{n}^{2}}\left(\phi_{n}\;\coth\;\phi_{n}-1\right)$$

where, *η* is effectiveness factor (0≤*η* ≤ 1), and *φ*_*n*_ is Thiele modulus. Rearrange Eq. (2) into Eq. (3):

(3) $$\eta \phi_{n}^{2}=3\left(\phi_{n}\;\coth\;\phi_{n}-1\right)$$

The left-hand side of Eq. (3) is the Weisz-Prater parameter which can be expressed as Eq. (4).

(4) $$\eta \phi_{n}^{2}=\frac{actualreactionrate}{adiffusionrate}=\frac{-r_{TG}^{\prime }\left(obs\right)\rho_{C}R^{2}}{D_{e}C_{As}}$$

where *–r*_*TG*_^*’*^*(obs)* is actual reaction rate of triglycerides species per catalyst mass (mol TG/g.s), *ρ*_*c*_ is catalyst pellet density (g/cm^3^), *R* is catalyst particle radius (cm), *D*_*e*_ is effective diffusivity (cm^2^/s), and *C*_*As*_ is concentration of triglycerides species (mol/cm^3^). Combining Eqs. (3) and (4) obtains Eq. (5).

(5) $$\frac{-r_{A}^{'}\left(obs\right)\rho_{C}R^{2}}{D_{e}C_{TGs}}=\eta \phi_{n}^{2}=3\left(\phi_{n}\;\coth\;\phi_{n}-1\right)$$

In this experimental works of effectivenes factor and Thiele modulus analyses, the forms of *ρ*_*c*_, *D*_*e*_, and *C*_*TG*_ can be removed due to similar conditions. Eq. (5) was applied to experimental runs 1 and 2 and then comparing them to obtain:

(6) $$\frac{-r_{A2}^{'}R_{2}^{2}}{-r_{A1}^{'}R_{1}^{2}}=\frac{\phi_{12}\;\coth\;\phi_{12}-1}{\phi_{11}\;\coth\;\phi_{11}-1}$$

Therefore, the Thiele modulus is expressed in Eq. (7).

(7) $$\phi_{1}=R\sqrt{\frac{-r_{A}^{\prime }\rho_{C}}{D_{e}C_{TGs}}}$$

The ratio of the Thiele modulus for runs 1 and 2 is:

(8) $$\frac{\phi_{11}}{\phi_{12}}=\frac{R_{1}}{R_{2}}$$

**Figure 3:**
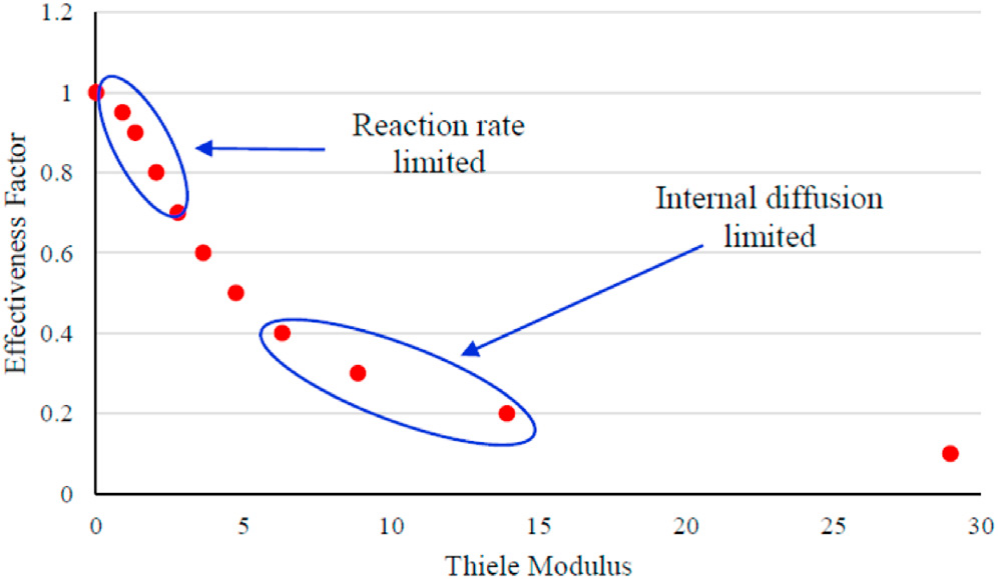
Relationship between effectiveness factors with Thiele modulus.

In order to make sure that the surface reaction is rate-limiting step, the effectiveness factor was chosen where the limiting step is the surface reaction (not internal diffusion limited) (Figure 3).

The value of the effectiveness factor was then substituted into Eq. (2) so that the value of Thiele modulus is obtained. The obtained Thiele modulus value was then entered into Eq. (8) so that the maximum radius of the catalyst particle was obtained.

### 2.7 Developing reaction rate law model and method for predicting reaction mechanism

#### 2.7.1 Development of reaction rate law model for the continuous flow hybrid catalytic-plasma reactor

**Figure 4:**
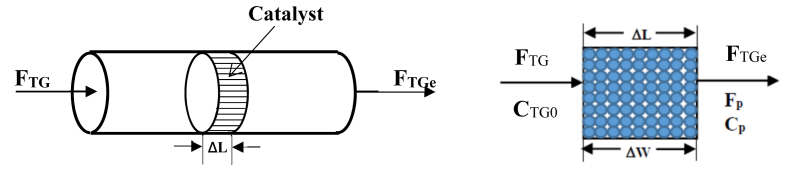
Differential reactor design in this kinetic study.

The reaction rate model was determined using an initial rate method as guided by Fogler (2006). The reactor is assumed to be a differential reactor in which the rate of reaction (-*r*_*TP*_) as function of concentration (*C*_*TG*_). For the differential reactor, conversion of the reactants (*X*_*TG*_) within the bed was assumed to be very small, so that the concentration of the reactants through the reactor bed is constant and close to the inlet reactant concentration. Therefore, the fixed bed reactor was assumed to be gradientless and the reaction rate was considered uniform throughout the bed. The differential reactor of the catalyst bed was depicted in Figure 4.

In methanolysis of vegetable oil for biodiesel production through the hybrid catalytic-plasma system, the catalytic transesterification reaction is assisted by plasma role or high energetic electrons to excite or even break down carbon-carbon bonds in triglyceride reactants on catalyst surface (Istadi et al., 2014; Lawson and Baosman, 2010). This hybrid catalysis-plasma role is assumed to be covered in the reaction rate constants in this study (constants parameter). Furthermore, the transesterification reaction follows Eq. (9):

(9) $$\text{TG}+3\text{M}\overset{{\text{k}}_{\text{s}}}{\underset{{\text{k}}_{\text{s}}^{'}}{⇄}}3\text{ME}+\text{G}$$

where TG is triglyceride, M is methanol, ME is methyl ester, and G is glycerol.

Calculation of reaction rate based on experimental data involves weight of catalyst (*ΔW*), volumetric feed flow rate of reactant (*υ*_*o*_), and concentration of reaction product (*C*_*ME*_). If *F*_*TGo*_ is the input flow rate and *F*_*TGe*_ is the output flow rate, the rate of reaction per unit mass of catalyst (*-r*_*TG*_^’^) at steady state can be calculated by Eq. (10):

(10) *F*_*TGo*_ - *F*_*TGe*_ + (*r*_*TG*_*’*) (*ΔW*) = 0

Solving for (-*r*_*TG*_^’^), it becomes:

(11) $$-r_{TG}^{\prime }=\frac{F_{TG_{o}}-F_{TG_{e}}}{\Delta W}$$

In terms of concentration, Eq. (11) can be written as:

(12) $$-r_{TG}^{\prime }=\frac{\upsilon_{0}C_{TG_{o}}-\upsilon C_{TG_{e}}}{\Delta W}$$

For constant volumetric flow rate, Eq. (12) reduces to:

(13) $$-r_{TG}^{\prime }=r_{ME}^{\prime }=\frac{\upsilon_{0}\left(C_{TG_{o}}-C_{TG_{e}}\right)}{\Delta W}=\frac{\upsilon_{0}C_{ME}}{\Delta W}$$

Concentration of methyl esters (*C*_*ME*_) was determined by the product composition analysis using GC-MS stated in Eq. (14), while the reaction rate (-*r’*_*TG*_) was calculated using Eq. (13). Meanwhile, yield of biodiesel was calculated using Eq. (15).

(14) $$C_{ME}=\frac{\rho_{biodiesel}}{MW_{biodiesel}}\;x\;\%ME_{GC\;Area}$$

(15) $$Yield\;of\;biodiesel=\frac{\rho_{biodiesel}xV_{biodiesel}}{weight\;of\;soybean\;oil\;}\;x\;100\text{\%}$$

#### 2.7.2 Predicting the reaction mechanism that appropriates to the experimental data (continuous flow hybrid catalytic-plasma reactor)

The reaction rate law model was predicted using the experimental data design method suggested by Fogler (2006) which based on intial rate. Design of feed concentration ratio variations on the experimental data was used to determine which reactant(s) is adsorbed on the catalyst surface during the catalytic reaction. This study was conducted by varying the molar ratio of methanol to oil (triglyceride) according to method suggested by Fogler (2006). In order to investigate dependence of reaction rate (-*r*_*TG*_) on the methanol reactant, the molar ratios of methanol:oil were varied as 3:1, 5:1, 7:1, and 17:1. Meanwhile, in order to investigate dependence of reaction rate (-*r*_*TG*_) on the triglyceride reactant, the molar ratios of methanol:oil were varied as 1:1/2, 1:1, 1:2, and 1:3. During the experiments, other process parameters, such as: voltage, WHSV, and diameter of catalyst pellet, were kept constant at 5 kV, 1.186 min^−1^, and 5 mm, respectively. Therefore, Table 1 describes reaction performance of the hybrid catalytic-plasma reactor.

The selection of the methanol to oil ratio was used to predict whether oil (triglyceride) is adsorbed on the surface of the catalyst or not adsorbed, as well as the methanol, as guided in reference (Fogler, 2006). This method is important to predict the possible reaction mechanism suitable with the experimental data (Eley Rideal or LHHW or others), which was then continued to final check what does control the regime of reaction, whether the adsorption of reactant, the surface or reaction, or the desorption of product.

The reaction rate law model was finally determined from the relationship between the initial concentration of the reactant/product and the reaction rate value obtained from experimental data. For example, if the concentration of methanol reactant is increased extremely, and gives significant and not linearly effect on the product reaction rate (*r*_*ME*_), so that the methanol reactant is assumed to be adsorbed on the catalyst surface. In other works, if it does not give significant effect on the product reaction, it is assumed that methanol is very weakly adsorbed (*K*_*ME*_*C*_*ME*_ is very small relative to 1) on catalyst surface or within bulk liquid phase. Other phenomena may be found during the interpretation of experimental works which have other possible meanings regarding whether the reactants or products are adsorbed or not.

## 3 Results and discussion

### 3.1 Characterization of catalyst

#### 3.1.1 X-ray diffraction characterization

**Figure 5:**
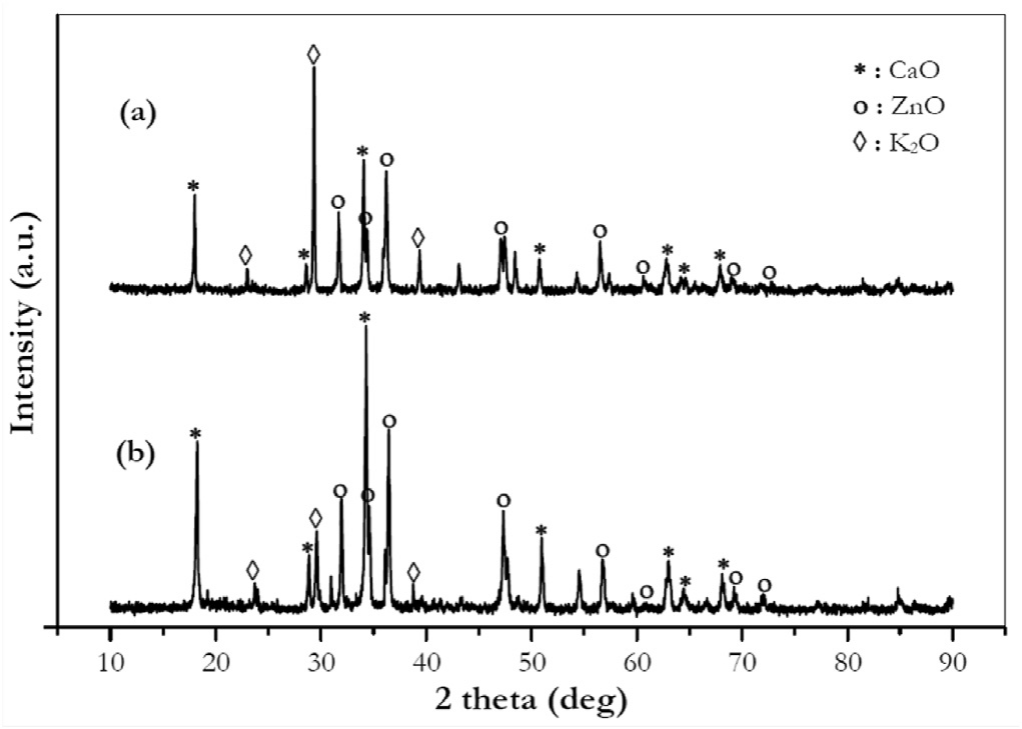
XRD pattern of the 5KCZ (K_2_O/CaO–ZnO) catalysts: (a) fresh, (b) spent.

Figure 5 illustrates the X-Ray Diffraction (XRD) pattern of a fresh and spent 5KCZ catalyst. The intensity of the peaks of CaO was shown at a 2θ angle of 17.8^o^, 28.6^o^, 33.6^o^, 50.7^o^, 63.2^o^, 64.2^o^ and 67.9^o^ (JCPDS File No. 37–1497). Meanwhile, the XRD pattern of ZnO was indicated at 31.7^o^, 34.4^o^, 36.2^o^, 47.5^o^, 56.5^o^, 62.8^o^, 69.02^o^, and 71.7^o^ (JCPDS File No. 36–1451). Whereas the peak intensity of K_2_O was exhibited at 23.04^o^, 29.4^o^, and 39.4^o^ (JCPDS File No. 47–1701; 77–2176). Figure 5(a) showed the high intensity peak of K_2_O. This was due to the impregnation of K_2_O on the surface of the CaO–ZnO catalyst. The peak intensity of K_2_O also showed that K_2_O was evenly dispersed on the surface of the catalyst. In Figure 5(b), the peak intensity of CaO and ZnO showed an increase, while the K_2_O intensity decreases. This decrease was due to the leaching process by methanol. However, the diffraction peaks of CaO, ZnO, and K_2_O on both catalysts indicate a similar pattern.

#### 3.1.2 Catalyst basicity

Table 2 shows the basicity of the catalyst before and after impregnation. The basicity of the catalyst increases after impregnation. The higher the basicity of the catalyst, the higher the catalyst activity (Kesić et al., 2012). High catalyst basicity increases triglyceride conversion as reported by Buchori et al. (2017). In this study, the impregnation of KNO_3_ on mixed metal oxides of CaO–ZnO was used to increase the basicity of the catalyst instead of decreased basicity due to the presence of ZnO and CaO components which were leached by methanol (Istadi et al., 2016). In addition, the catalyst impregnation with KNO_3_ also acts as a binder to increase the mechanical strength of the catalyst.

**Table 2:** Effect of K_2_O impregnation on catalyst basicity.

Catalyst	Basicity (mmol/gram)
CaO–ZnO	0.662
5KCZ (fresh)	1.090
5KCZ (spent)	1.00

Table 2 also indicates a slight decrease in basicity of the 5KCZ catalyst after the transesterification process (catalyst spent). This fact is also shown by Figure 5 where the intensity peak of K_2_O also decreases after it is used for the transesterification process. The decrease of basicity was mainly due to CaO leaching by methanol rather than K_2_O loss. Research conducted by Istadi et al. (2016) and Taufiq-Yap et al. (2011) proved that the CaO content decreased after the transesterification process was carried out due to leached by methanol.

### 3.2 Effect of plasma role on the transesterification process over the continuous flow hybrid catalytic-plasma reactor

The use of plasma role in the hybrid catalytic-plasma reactor gives significant effect on the transesterification process to produce biodiesel. This effect of plasma was investigated by comparing the catalytic transesterification process with or without plasma as presented in Table 3.

**Table 3:** Role of plasma assistance on the catalytic transesterification process of methanol and triglyceride to produce biodiesel plasma.

Treatments	Yield of biodiesel (%)	Space time (min)
Catalytic transesterification without plasma^(a)^	49.8	1.25
Catalytic transesterification using plasma (hybrid) ^(b)^	77.2	1.25

(a) Condition: methanol:oil ratio of 15:1, voltage of 1.5 kV, WHSV of 1.186/min, and diameter of catalyst pellet of 5 mm.

(b) Condition: methanol:oil ratio of 15:1, WHSV of 1.186/min, and diameter of catalyst pellet of 5 mm.

The transesterification process was carried out using the catalyst of 5% K_2_O/CaO–ZnO with space time of 1.25 min Table 3 shows that transesterification with plasma (hybrid catalytic-plasma reactor) demonstrating significantly better results (biodiesel yield of 77.2%) rather than when one without plasma (biodiesel yield of 49.8%). From this result, it is suggested that the transesterification is assisted by plasma role, where the catalyst and the plasma play simultaneous roles in improving the biodiesel production. The promising effect is also indicated by reducing the space time (like reaction time in batch reactor) significantly from usually 1–2 h to only 1.25 min due to the significant role of plasma effect (Table 3). In the hybrid catalytic plasma, the high voltage applied to the electrode plays a role in providing high energetic electrons on assisting the catalytic reaction in surface of catalyst. The high energetic electrons from plasma may excite the electron pairs in covalent bonding of reactant molecules in the discharge zone. The excitation phenomenon occurs may be due to higher energy, brought by electrons from the high voltage electrode, than the bonding energy of reactant molecules. Therefore, the catalyst placed in the plasma discharge zone has role on reducing activation energy required for the transesterification reaction and is assisted by the presence of high-energy electrons from plasma role on the catalyst surface. Indeed, the reaction between an excited atom or molecule and the other reactant molecule on the catalyst surface will be faster significantly.

### 3.3 Analysing the Thiele Modulus and effectivenes factor on maintaining the surface reaction rate limiting step

Analysing the Thiele modulus and effectivenes factor was determined by comparing two different size of catalyst pellets (0.0035 and 0.0025 m) in order to make sure that the reaction rate study is within the surface reaction rate limiting step. The experimental works related to this analysis were carried out under the same operating conditions as given in Table 4. The 15.15 g of catalyst pellets were placed into discharge zone of the hybrid catalytic-plasma reactor. The space time was very short at 1.25 min so that the external mass transfer resistance was assume to be ignored.

**Table 4:** Measured reaction rates on different size catalyst pellets.

Run	Catalyst pellet radius (m)	$r_{ME}^{{\;}^{\prime }}\left(\frac{gmol_{ME}}{gcat.s}\right)$
1	0.0035	6.74 × 10^−5^
2	0.0025	7.05 × 10^−5^

The Thiele modulus for run 1 was compared to run 2 according to Eq. (8) giving: *φ*_*11*_ *=* 1.4*φ*_*12*_. Substituting φ_11_ in Eq. (6) and evaluating –*r*_*TG*_′ and *R* for runs 1 and 2 gave:

(16) $$0.54=\frac{\phi_{12}\;\coth\;\phi_{12}-1}{1.4\phi_{12}\;\coth\;\left(1.4\phi_{12}\right)-1}$$

Solving Eq. (16) to obtain:

*φ*_*12*_ = 0.89 for *R*_*2*_ = 0.0025 m

*φ*_*11*_ = 1.4 and *φ*_*12*_ = 1.26 for *R*_*1*_ = 0.0035 m

Substituting *φ*_*11*_ and *φ*_*12*_ in Eq. (2) to obtain effectiveness factor as follow:

For *R*_*2*_: *η*_*2*_ = 0.95

For *R*_*1*_: *η*_*1*_ = 0.91

To ignore internal diffusion control, the particle radius (*R*) was calculated by introducing the effectiveness factor into Eq. (2) (example, *η* = 0.95). Therefore, the Thiele Modulus was:

(17) $$0.95=\frac{3\left(\phi_{13}\;\cot\text{h}\;\phi_{13}-1\right)}{\phi_{13}^{2}}$$

*φ*_*13*_ = 0.90

The maximum radius or diameter of particle was obtained by comparing the value of Thiele Modulus in Eq. (8), as follow:

(18) $$R_{2}=R_{1}\frac{\phi_{13}}{\phi_{11}}=\left(0.0035\right)\frac{0.90}{1.26}=0.0025\;m=2.5\;mm$$

The effectiveness factor analysis result indicates that the maximum radius of catalyst particle needed to ignore the internal diffusion control is 2.5 mm (or 5 mm diameter), so that the reaction mechanism is only controlled by catalyst surface reaction or within chemical reaction regimes or not a diffusion regimes.

### 3.4 Developing reaction rate law model appropriate to the plasma-assisted catalytic transesterification process over the continuous flow hybrid catalytic-plasma reactor

Table 2 shows increasing Fatty Acid Alkyl Esters (FAAE) product reaction rates (-*r’*_*TG*_ *= r’*_*ME*_) in runs number 1, 2, and 3 when the methanol concentration is increased, but it is not linear. Likewise in the runs 3 and 4 (Table 1), when the methanol concentration is increased extremely, while the biodiesel reaction rate (-*r’*_*TG*_) also increases sharply. This facts show a proportional relation between the initial concentration of reactant (methanol) and the increasing reaction rate (-*r’*_*TG*_) which indicates that methanol is adsorbed on the catalyst surface. With respect to determining whether the triglycerides are adsorbed on the catalyst surface or not, it can be suggested from the results of runs 5 and 8 (Table 1) at constant methanol. From the results, when the triglyceride concentration increases, the FAAE reaction rate (-*r’*_*TG*_) also increases. However, as shown in runs number 6 and 7 (Table 1), when the triglyceride concentration raises linearly, the resulting FAAE reaction rate (-*r’*_*TG*_) increases sharply. Furthermore, as shown in runs number 7 and 8 (Table 1), the triglyceride concentration increases, but the FAAE reaction rate actually decreases. This facts indicate non-linearly relation between the initial concentration of triglyceride and the rate of reaction (-*r’*_*TG*_) suggesting that the triglyceride is not adsorbed on the catalyst surface during the reaction.

Therefore, from the results, it can be concluded that the possible reaction mechanism on this plasma-assisted transesterification reaction over the continuous flow hybrid catalytic-plasma reactor follows the classical Eley-Rideal mechanism, where one of reactant is adsorbed on the catalyst surface and another one is in bulk phase (not adsorbed). Result of reaction kinetic model test of transesterification reaction at hybrid catalytic-plasma reactor was described in Table 5. After the possible reaction mechanisms were found, the reaction rate law model (-*r*_*TG*_ = *r*_*ME*_ *= r*_*s*_) was proposed according to the predicted mechanism (Eq. (19)).

(19) $$\left(r_{s}\right)=\frac{k_{s}\left(C_{TG}K_{M}C_{M}^{3}-\frac{K_{ME}}{K_{s}}C_{ME}^{3}C_{G}\right)}{{\left(K_{M}^{\frac{1}{3}}C_{M}+K_{ME}^{\frac{1}{3}}C_{ME}+1\right)}^{3}}$$

where *C*_*TG*_, *C*_*ME*_, *C*_*M*_, and *C*_*G*_ represent concentrations of triglyceride, methyl ester, methanol and glycerol, respectively. Meanwhile, *k*_*s*_, *K*_*M*_, *K*_*ME*_, and *K*_*s*_ are reaction rate constant, equilibrium adsorption constants of methanol and methyl ester, and equilibrium constant of surface reaction, respectively. Thus, the most possible reaction rate law model was fitted to the experimental data (Table 1) to determine the model parameter, i.e. *k*_*s*_, *K*_*M*_, *K*_*ME*_, and *K*_*s*_ (Eq. (19)). The nonlinear fitting was conducted using the Polymath Pro 6.0 software. We selected the reaction rate law model that having closest R^2^ to 1 and/or the highest F-value.

**Table 5:** Result of reaction kinetic model test of transesterification reaction at hybrid catalytic-plasma reactor.

No.	Prediction of Reaction Mechanism	Reaction Kinetic Model (mole/gcat.min)	Value of Reaction Rate Constant	R^2^ Value Fischer F-Value (ANOVA)
I	**The reaction of TG with adsorbed M gives ME and G** $\text{TG}+3\text{Ms}\overset{{\text{k}}_{\text{s}}}{\underset{{\text{k}}_{\text{s}}^{\prime }}{⇄}}3\text{ME}+\text{G}+3\text{s}$ The reaction mechanism is as follows: 1) 3M+3s⇌3Ms 2) TG+3Ms⇌3ME+G+3s	(rs)=ks(CTGKMCM3−CME3CGKs)(KM13CM+1)3	*k*_*s*_ = 0.0147 *K*_*M*_ = 0.0026 *K*_*s*_ = 0.0101	R^2^ = 0.8816 F-value = 14.6785
II	**The reaction of TG with adsorbed M gives desorbed ME and G** $\text{TG}+3\text{Ms}\overset{{\text{k}}_{\text{s}}}{\underset{{\text{k}}_{\text{s}}^{\prime }}{⇄}}3\text{MEs}+\text{G}$ The reaction mechanism is as follows: 1) 3M+3s⇌3Ms 2) TG+3Ms⇌3MEs+G 3) 3MEs⇌3ME+3s	(rs)=ks(CTGKMCM3−KMEKsCME3CG)(KM13CM+KME13CME+1)3	*k*_*s*_ = 0.0078 *K*_*M*_ = 0.0061 *K*_*ME*_ = 2.942 × 10^−6^ *K*_*s*_ = 0.9709	R^2^ = 0.9267 F-value = 22.8575
III	**The reaction of TG with adsorbed M gives ME and G desorbed** $\text{TG}+3\text{Ms}\overset{{\text{k}}_{\text{s}}}{\underset{{\text{k}}_{\text{s}}^{\prime }}{⇄}}3\text{ME}+\text{Gs}+2\text{s}$ The reaction mechanism is as follows: 1) 3M+3s⇌3Ms 2) TG+3Ms⇌3ME+Gs+2s 3) Gs⇌G+s	(rs)=ks(CTGKMCM3−KGKsCME3CG)(KM13CM+KGCG+1)3	*k*_*s*_ = 0.0035 *K*_*M*_ = 0.0418 *K*_*G*_ = 0.4189 *K*_*s*_ = 1.1009	R^2^ = 0.5912 F-value = 2.1336
IV	**The reaction of TG with adsorbed M gives desorbed ME and desorbed G** $\text{TG}+3\text{Ms}+\text{s}\overset{{\text{k}}_{\text{s}}}{\underset{{\text{k}}_{\text{s}}^{\prime }}{⇄}}3\text{MEs}+\text{Gs}$ The reaction mechanism is as follows: 1) 3M+3s⇌3Ms 2) TG+3Ms+s⇌3MEs+Gs 3) 3MEs⇌3ME+3s 4) Gs⇌G+s	(rs)=ks(CTGKMCM3−KMEKGKsCME3CG)(KM13CM+KME13CME+KGCG+1)4	Result: Divergence	-

**Annotation**: TG = triglyceride; M = methanol; ME = methyl ester; G = glycerol; TGs = adsorbed triglyceride; Ms = adsorbed methanol; MEs = adsorbed methyl ester; Gs = adsorbed glycerol; C_TG_ = concentration of triglyceride (gmol/L); C_M_ = concentration of methanol (gmol/L); C_ME_ = concentration of methyl ester (gmol/L); C_G_ = concentration of glycerol (gmol/L); k_s_ = constant of reaction rate for surface reaction, (min^−1^); k_M_ = constant of reaction rate for M component (methanol), (min^−1^); k_ME_ = constant of reaction rate for ME component (methyl ester), (min^−1^); k_G_ = constant of reaction rate for G component (glycerol), (min^−1^); K_s_ = equilibrium constant for surface reaction; K_M_ = equilibrium constant for M component (methanol); K_ME_ = equilibrium constant for ME component (methyl ester); K_G_ = equilibrium constant for G component (glycerol).

Plasma, in the transesterification reaction, produces species excited by high-energy electrons, including vibrationally-/electronically excited, ionized, and radical species. Excited species have a greater energy level than species in the ground state. As a result, the interaction between the excited species and the catalyst surface will decrease the activation energy (Kim et al., 2017). Role of high energetic electrons from plasma on the reaction rate is covered in *k*_*s*_ parameter, where the electrons attack C–O bonding of triglyceride (Eq. (23)). Thus, final consideration of fitting the reaction rate law model on the experimental data was not only based on the value of determinatiion coefficient (R^2^) and Fischer value (*F*-value), but also considering some phenomenological reasons as stated in previous section. According to the reaction rate model following Eley-Rideal model/mechanism stated in Eq. (19) (Al-Sakkari et al., 2017; Dossin et al., 2006; Kapil et al., 2011; Muciño et al., 2016; Xiao et al., 2010) fitted to experimental data (Table 1), the reaction rate law model for the plasma-assisted transesterification over Hybrid Catalytic-Plasma reactor is resulted in Eq. (20). Fitness of the reaction rate law model on the experimental data (Table 1) was based on coefficient of determination (R^2^) and Fischer (*F*) value of Analysis of Variance (ANOVA).

(20) $$-r_{TG}=r_{ME}=r_{s}=\frac{0.0078\left(0.0061C_{TG}C_{M}^{3}-3.0302x10^{-6}C_{ME}^{3}C_{G}\right)}{{\left(0.1827C_{M}+0.0145C_{ME}+1\right)}^{3}}$$

### 3.5 Prediction of reaction mechanism of the plasma-assisted catalytic transesterification process over the continuous flow hybrid catalytic-plasma reactor

Possible reaction mechanism of the plasma-assisted catalytic transesterification process in a continuous hybrid catalytic-plasma reactor was suggested based on the deduction of reaction rate law from the experimental reaction data as explained in previous section. It was suggested that the reaction rate law model follows Eley-Rideal mechanism in which methanol is adsorbed on the catalyst surface and reacts with triglyceride in bulk phase to produce adsorbed methyl/alkyl ester on the catalyst surface and glycerol. The predicted reaction mechanism of FAME/FAAE formation in the plasma assisted-transesterification reaction within the continuous hybrid catalytic-plasma reactor can be described as follows:

STEP 1 Adsorption of methanol on the catalyst surface (Eq. (21)): The O group of –OCH_3_ is attracted into the K group on the catalyst surface due to the K group is more reactive than the O group.(21)
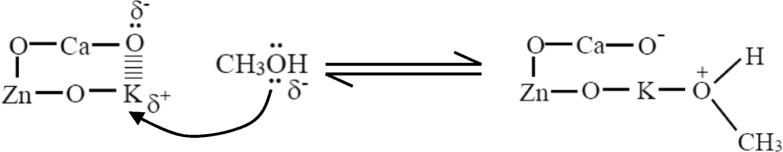

The H atom of methanol is attracted by the O atom at the catalyst surface as illustrated in Eq. (22).(22)
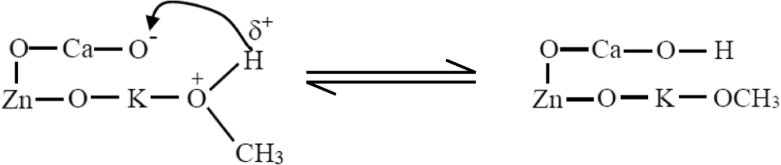

STEP 2 High energetic electrons from the high voltage electrode collide with reactant molecules and disrupt the electron pairs of the reactant covalent bonds of the O–C in the triglyceride or excite the pair electrons in the covalent bond. This abstraction role of plasma electrons was included in *k*_s_ of the reaction rate law model (Eq. (19)). The plasma electrons energy is quite higher than the chemical bond energy in the triglyceride, so that the chemical bonds of these electron pairs are readily excited or even broken which further form carbonated carbonyls as depicted in Eq. (23). This reaction occurs very fast.(23)
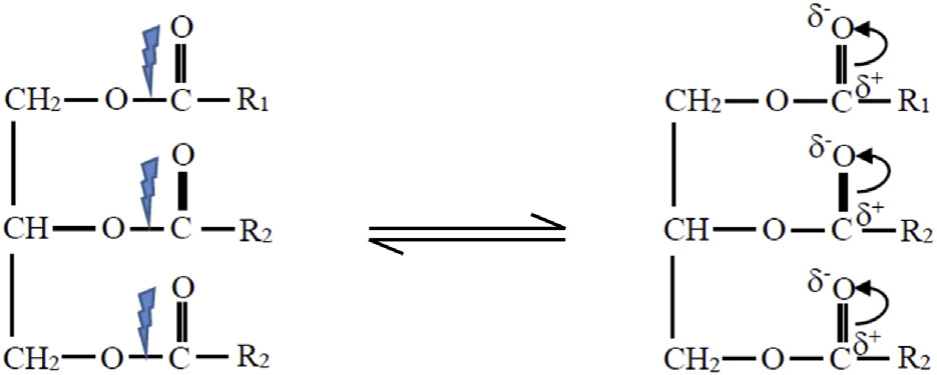

STEP 3 The transesterification reaction occurs on the catalyst surface. The carbon carbonyl from excited triglyceride molecules becomes so weak that can readily react with the methoxide anion from the surface of the O–Ca–O–Zn–O–K catalyst to form tetrahedral intermediate (Eq. (24)). Furthermore, the catalyst decreases the activation energy of this reaction and the reaction becomes faster.(24)
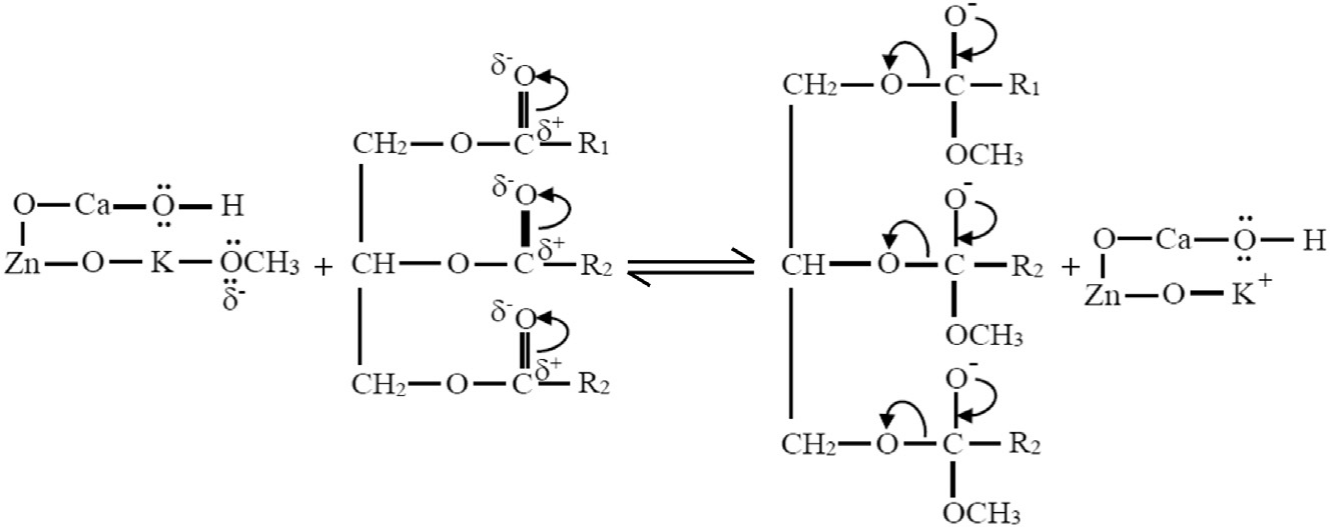

STEP 4 Tetrahedral intermediates rapidly breaks down into fatty acid alkyl esters and glycerol anions (Eq. (25)). The plasma process is very fast, while the catalytic process also occurs more rapidly. The synergism of these two processes produced a benefit of very short time required for the transesterification reaction significantly.(25)
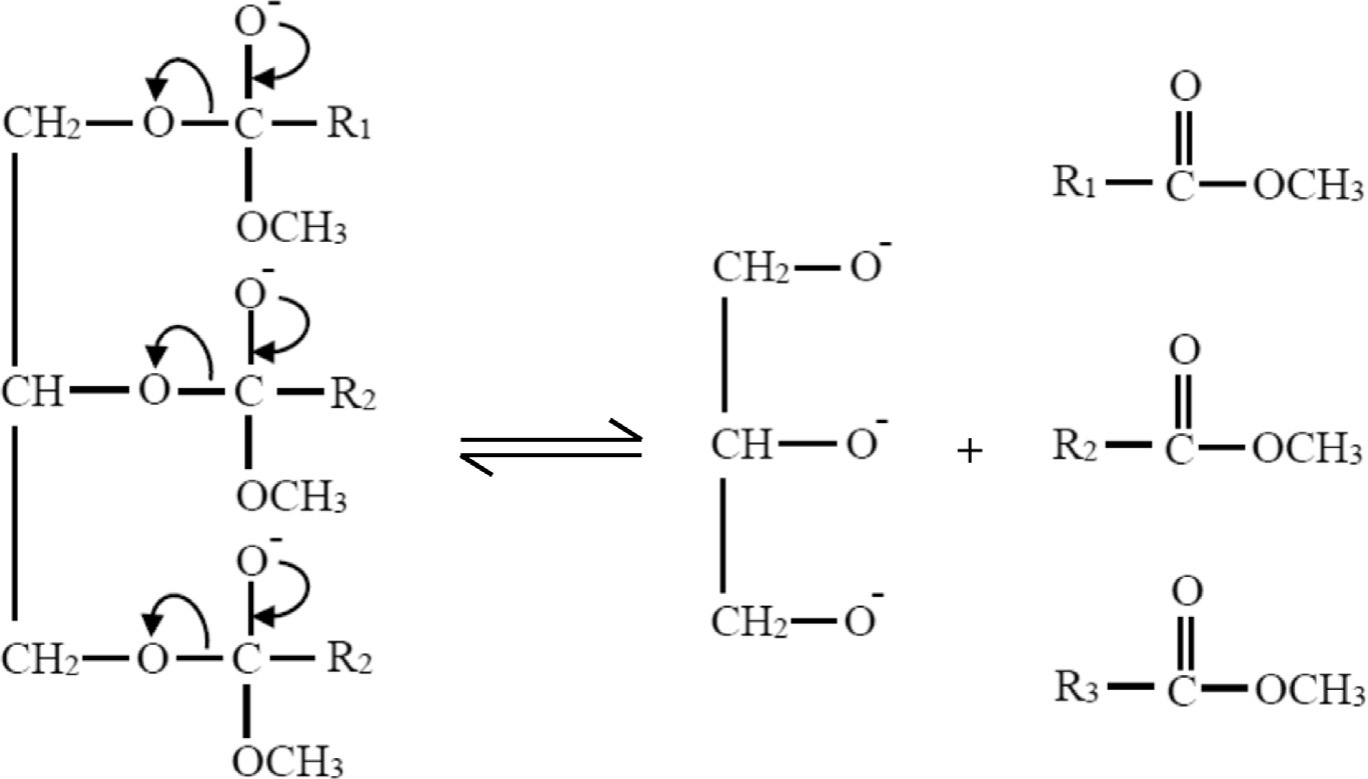

STEP 5 In the final steps, the glycerol anion takes the H ions from the catalyst surface to form glycerol (Eq. (26)).(26)
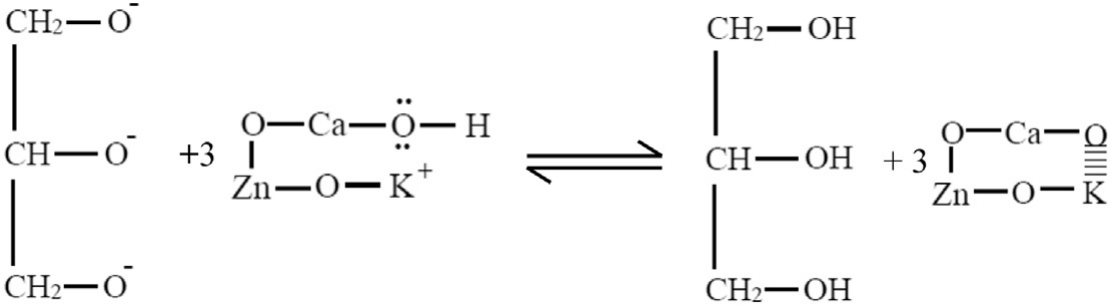

### 3.6 Utilization of the resulted reaction rate law model for designing a continuous flow hybrid catalytic-plasma reactor

The final reaction rate law model in Eq. (20) can be used to design a continuous hybrid catalytic-plasma (fixed bed) reactor. The found reaction rate equation is used to find the relationship between reactant conversion (*X*_*A*_) to be achieved and space time (τ) required for the reaction, which means it is possible to predict the space time required to produce maximum conversion. The relationship between conversion (*X*_*A*_) and space time (τ) can be obtained from the mass balance equation as follows:

(27) $$F_{A}|W-F_{A}|W+\Delta W+r_{A}\Delta W=0$$

(28) $$\frac{dF_{A}}{dW}=-r_{A}$$

(29) *dF*_*A*_ = *F*_*A0*_*dX*_*A*_ = *C*_*A0*_*υ*_*0*_*dX*_*A*_

(30) *dW = ρ*_*b*_*dV = ρ*_*b*_*υ*_*0*_*dτ*

**Figure 6:**
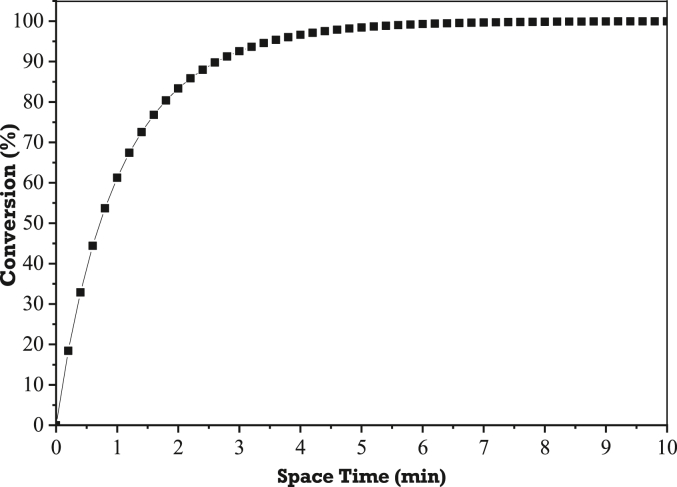
The relationship between reactant conversion (*X*_*A*_) to be achieved and space time (τ) required for the transesterification reaction in the continuous hybrid catalytic-plasma reactor.

Eq. (27) is rearranged to become Eq. (31):

(31) $$\frac{dX_{A}}{d\tau }=\frac{\rho_{b}}{C_{A0}}\left(-r_{A}\right)$$

where τ is space time (minute), *V* is reactor volume (L), υ_0_ is volumetric flow rate (L/min), ρ_b_ is catalyst bulk density in the reactor (g/L), W is catalyst weight (g), and *C*_*A0*_ is initial concentration of triglyceride (mol/L). Eq. (14) is substituted in Eq. (31) which results in the relationship between conversion and space time as shown in Figure 6. In this reactor design, the space time (τ) represents the length of fixed bed reactor required. Figure 6 shows that the maximum equilibrium triglyceride conversion that can be achieved when using the continuous hybrid catalytic-plasma reactor is 99% with a space time required only 5 min. At this condition, the required reactant feed flow rate is 5.66 mL/min with a WHSV of 0.299 min^−1^. These results prove that the hybrid catalytic reactor plasma is capable for producing high reactant conversion when the transesterification reaction is carried out under the optimal operating conditions.

## 4 Conclusion

It was found that plasma has significant role on the catalytic transesterification reaction in a continuous flow hybrid catalytic-plasma reactor especially biodiesel yield and reaction/space time. Yield of biodiesel produced over the plasma-assisted transesterification was higher significantly than the transesterification without plasma. Meanwhile, the reaction time required or space time over the continuous flow hybrid catalytic-plasma reactor is very short in compared to the conventional reactor. With respect to kinetic study, the plasma assisted transesterification reaction over the continuous flow hybrid catalytic-plasma reactor follows the Eley-Rideal mechanism model, where the methanol adsorbed on the catalyst surface reacts with triglycerides in bulk phase to produce an adsorbed methyl ester and glycerol in bulk phase. According to this reaction mechanism, the possible reaction rate law model found is:

$$-r_{TG}=r_{ME}=r_{s}=\frac{0.0078\left(0.0061C_{TG}C_{M}^{3}-3.0302x10^{-6}C_{ME}^{3}C_{G}\right)}{{\left(0.1827C_{M}+0.0145C_{ME}+1\right)}^{3}}{\text{gmol.gcat}}^{-1}{\text{.min}}^{-1}.$$

Using this reaction rate law model appropriate to the plasma-assisted catalytic transesterification over hybrid catalytic-plasma reactor, it is possible to design the length of fixed bed or hybrid catalytic-plasma reactor required for the reaction by utilizing the relationship between conversion and space time. In this reactor design, the space time represents the length of required fixed bed reactor. Finally, the possible maximum conversion of triglyceride of 99% can be achieved with a space time required only 5 min when using the continuous flow hybrid catalytic-plasma reactor. At this condition, the required reactant feed flow rate is 5.66 mL min^−1^ with a WHSV of 0.299 min^−1^.

## Declarations

### Author contribution statement

P. Purwanto: Analyzed and interpreted the data; Wrote the paper.

Luqman Buchori: Conceived and designed the experiments; Performed the experiments; Analyzed and interpreted the data; Wrote the paper.

I. Istadi: Conceived and designed the experiments; Performed the experiments; Analyzed and interpreted the data; Contributed reagents, materials, analysis tools or data; Wrote the paper.

### Funding statement

This work was supported by the Research Institution and Community Service, 10.13039/501100005844Diponegoro University, Semarang, Indonesia under the research project of Riset Publikasi Internasional Bereputasi Tinggi (RPIBT) with contract number: 387-07/UN7.P4.3/PP/2018 Year 2018–2020.

### Competing interest statement

The authors declare no conflict of interest.

### Additional information

No additional information is available for this paper.
